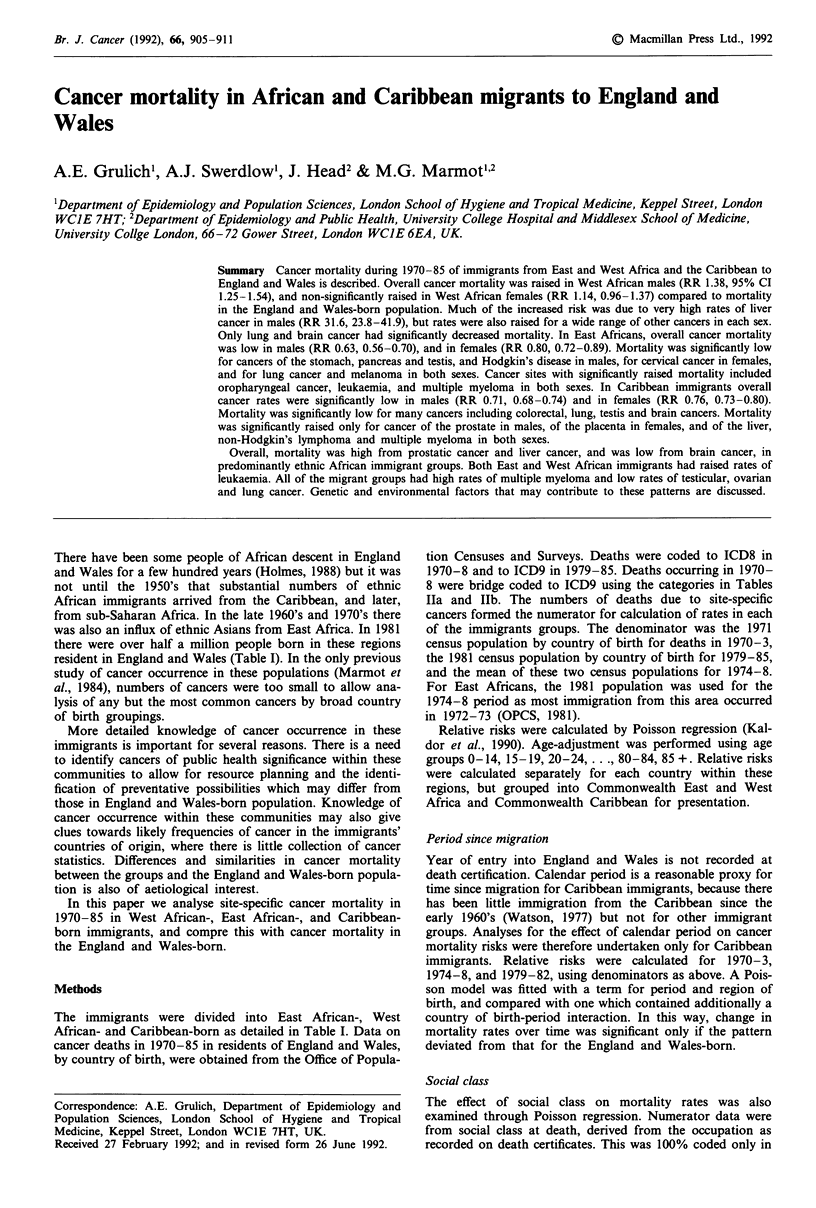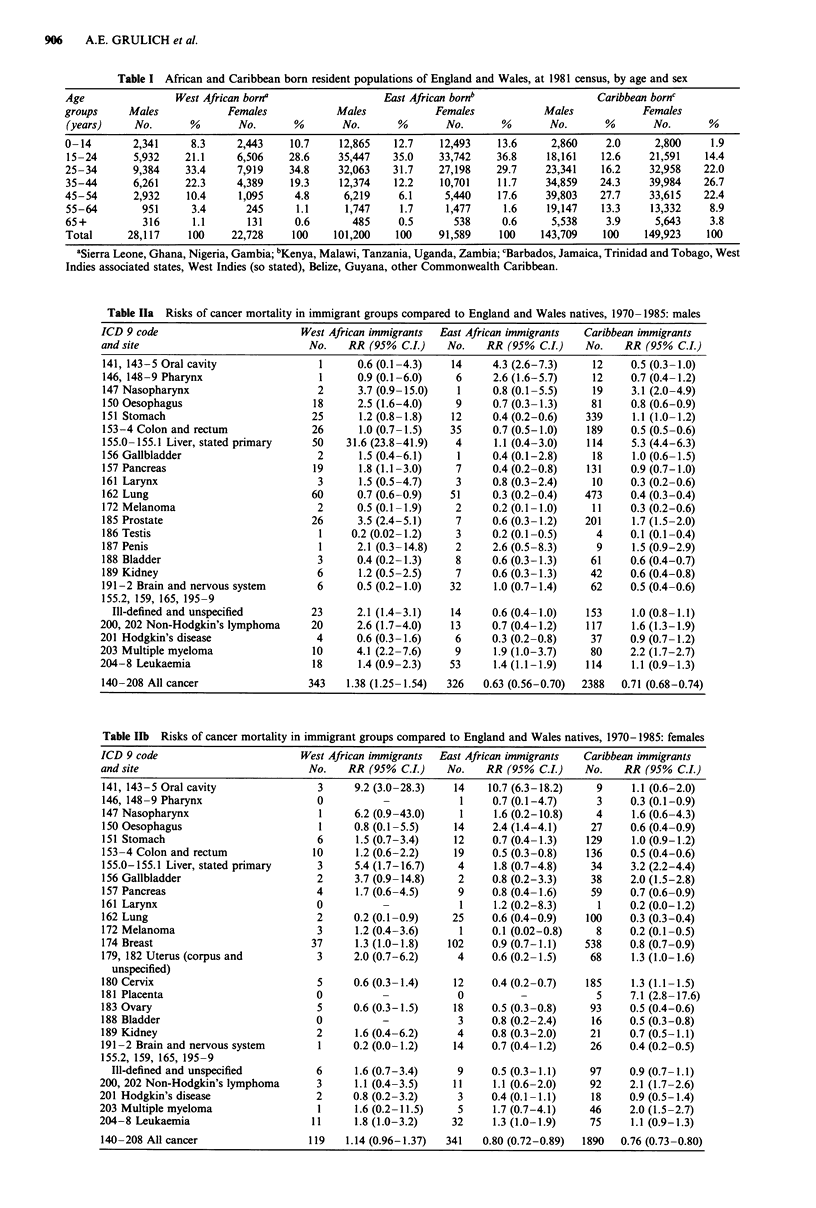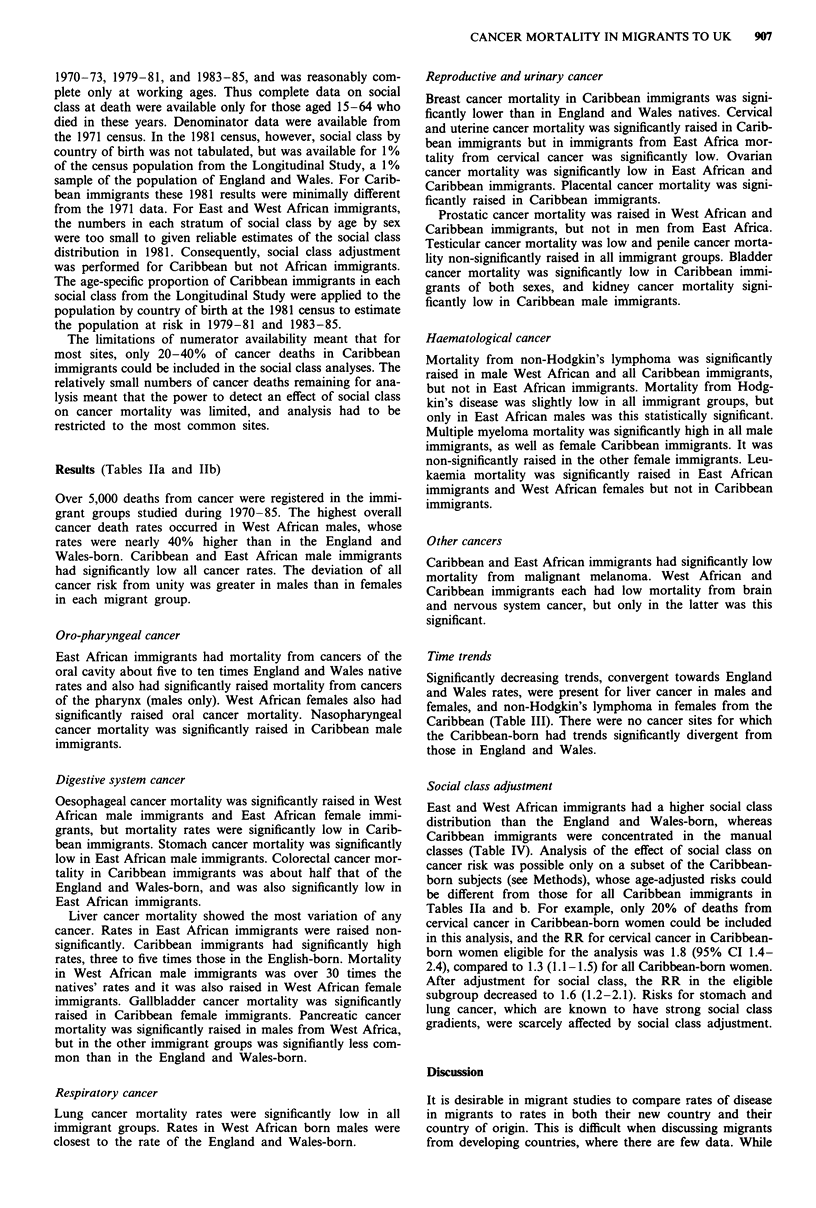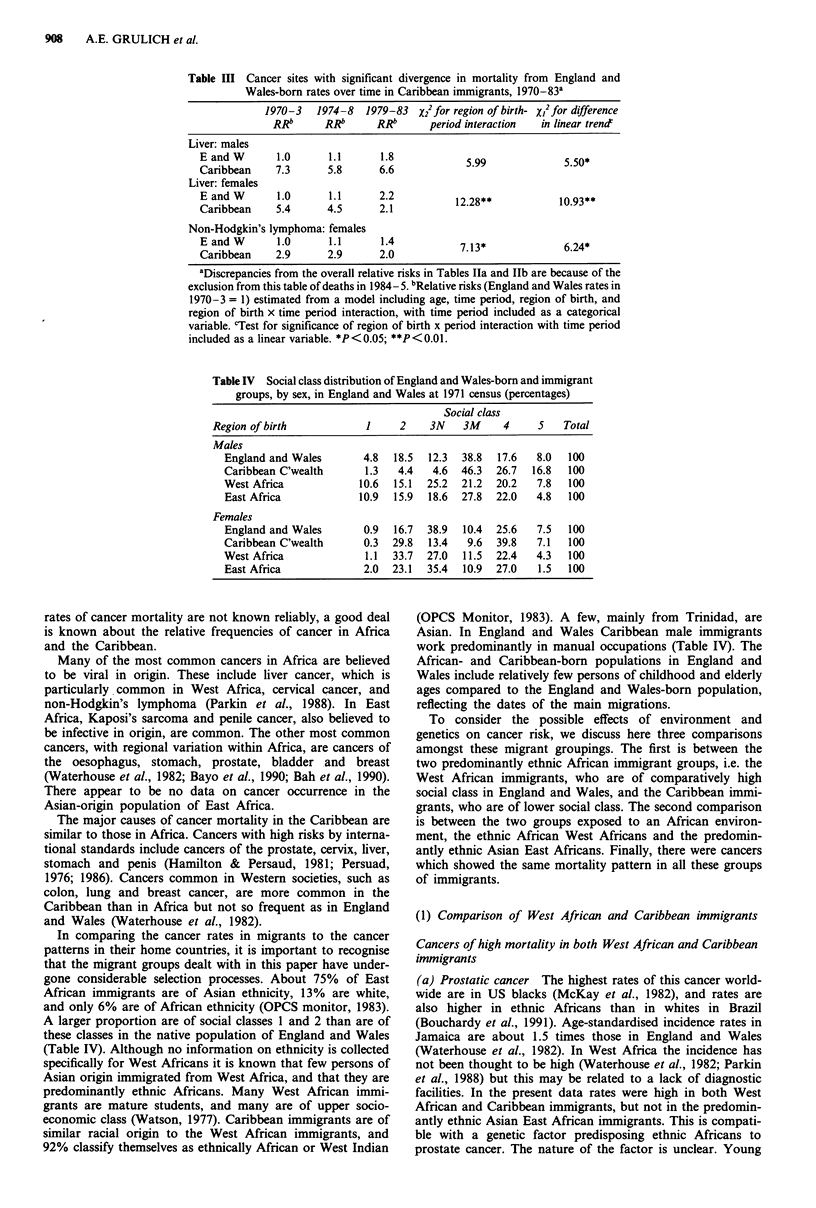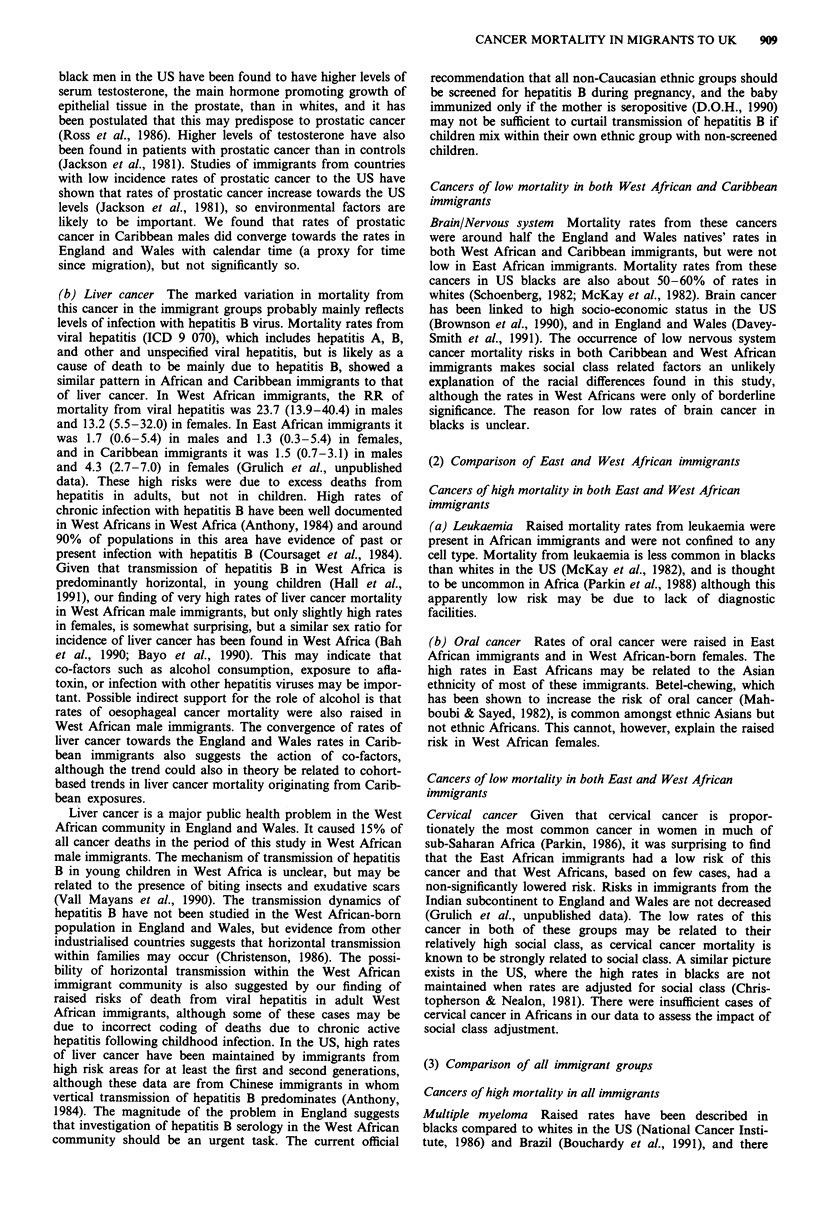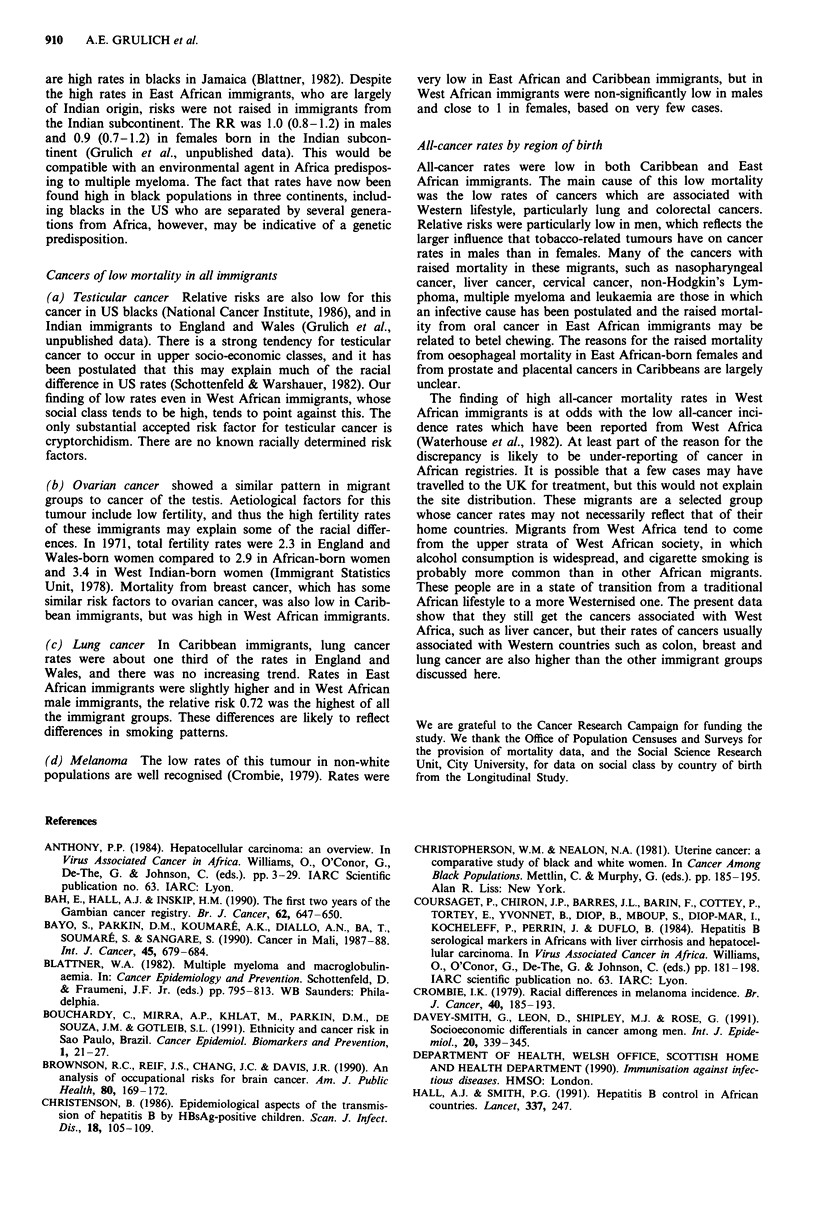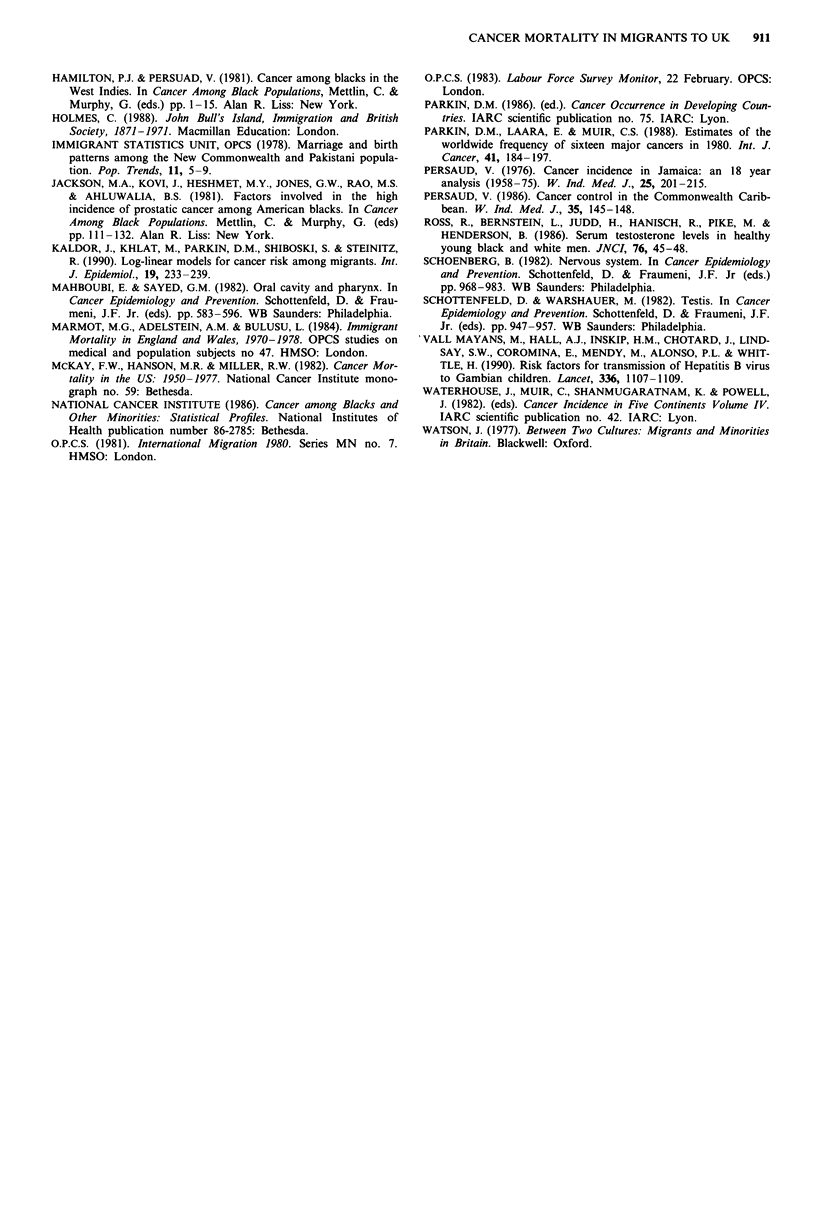# Cancer mortality in African and Caribbean migrants to England and Wales.

**DOI:** 10.1038/bjc.1992.383

**Published:** 1992-11

**Authors:** A. E. Grulich, A. J. Swerdlow, J. Head, M. G. Marmot

**Affiliations:** Department of Epidemiology and Population Sciences, London School of Hygiene and Tropical Medicine, UK.

## Abstract

Cancer mortality during 1970-85 of immigrants from East and West Africa and the Caribbean to England and Wales is described. Overall cancer mortality was raised in West African males (RR 1.38, 95% CI 1.25-1.54), and non-significantly raised in West African females (RR 1.14, 0.96-1.37) compared to mortality in the England and Wales-born population. Much of the increased risk was due to very high rates of liver cancer in males (RR 31.6, 23.8-41.9), but rates were also raised for a wide range of other cancers in each sex. Only lung and brain cancer had significantly decreased mortality. In East Africans, overall cancer mortality was low in males (RR 0.63, 0.56-0.70), and in females (RR 0.80, 0.72-0.89). Mortality was significantly low for cancers of the stomach, pancreas and testis, and Hodgkin's disease in males, for cervical cancer in females, and for lung cancer and melanoma in both sexes. Cancer sites with significantly raised mortality included oropharyngeal cancer, leukaemia, and multiple myeloma in both sexes. In Caribbean immigrants overall cancer rates were significantly low in males (RR 0.71, 0.68-0.74) and in females (RR 0.76, 0.73-0.80). Mortality was significantly low for many cancers including colorectal, lung, testis and brain cancers. Mortality was significantly raised only for cancer of the prostate in males, of the placenta in females, and of the liver, non-Hodgkin's lymphoma and multiple myeloma in both sexes. Overall, mortality was high from prostatic cancer and liver cancer, and was low from brain cancer, in predominantly ethnic African immigrant groups. Both East and West African immigrants had raised rates of leukaemia. All of the migrant groups had high rates of multiple myeloma and low rates of testicular, ovarian and lung cancer. Genetic and environmental factors that may contribute to these patterns are discussed.


Br. J. Cancer (1992), 66, 905-911 © Macmillan Press Ltd., 1992

### Cancer mortality in African and Caribbean migrants to England and Wales

A.E. Grulich[1], A.J. Swerdlow[1], J. Head[2] & M.G. Marmot[1,2]

[1]Department of Epidemiology and Population Sciences, London School of Hygiene and Tropical Medicine, Keppel Street, London WC1E 7HT; [2]Department of Epidemiology and Public Health, University College Hospital and Middlesex School of Medicine, University Collge London, 66-72 Gower Street, London WC1E 6EA, UK.

**Summary** Cancer mortality during 1970-85 of immigrants from East and West Africa and the Caribbean to England and Wales is described. Overall cancer mortality was raised in West African males (RR 1.38, 95% CI 1.25-1.54), and non-significantly raised in West African females (RR 1.14, 0.96-1.37) compared to mortality in the England and Wales-born population. Much of the increased risk was due to very high rates of liver cancer in males (RR 31.6, 23.8-41.9), but rates were also raised for a wide range of other cancers in each sex. Only lung and brain cancer had significantly decreased mortality. In East Africans, overall cancer mortality was low in males (RR 0.63, 0.56-0.70), and in females (RR 0.80, 0.72-0.89). Mortality was significantly low for cancers of the stomach, pancreas and testis, and Hodgkin's disease in males, for cervical cancer in females, and for lung cancer and melanoma in both sexes. Cancer sites with significantly raised mortality included oropharyngeal cancer, leukaemia, and multiple myeloma in both sexes. In Caribbean immigrants overall cancer rates were significantly low in males (RR 0.71, 0.68-0.74) and in females (RR 0.76, 0.73-0.80). Mortality was significantly low for many cancers including colorectal, lung, testis and brain cancers. Mortality was significantly raised only for cancer of the prostate in males, of the placenta in females, and of the liver, non-Hodgkin's lymphoma and multiple myeloma in both sexes.

Overall, mortality was high from prostatic cancer and liver cancer, and was low from brain cancer, in predominantly ethnic African immigrant groups. Both East and West African immigrants had raised rates of leukaemia. All of the migrant groups had high rates of multiple myeloma and low rates of testicular, ovarian and lung cancer. Genetic and environmental factors that may contribute to these patterns are discussed.

There have been some people of African descent in England and Wales for a few hundred years (Holmes, 1988) but it was not until the 1950's that substantial numbers of ethnic African immigrants arrived from the Caribbean, and later, from sub-Saharan Africa. In the late 1960's and 1970's there was also an influx of ethnic Asians from East Africa. In 1981 there were over half a million people born in these regions resident in England and Wales (Table I). In the only previous study of cancer occurrence in these populations (Marmot *et al.*, 1984), numbers of cancers were too small to allow analysis of any but the most common cancers by broad country of birth groupings.

More detailed knowledge of cancer occurrence in these immigrants is important for several reasons. There is a need to identify cancers of public health significance within these communities to allow for resource planning and the identification of preventative possibilities which may differ from those in England and Wales-born population. Knowledge of cancer occurrence within these communities may also give clues towards likely frequencies of cancer in the immigrants' countries of origin, where there is little collection of cancer statistics. Differences and similarities in cancer mortality between the groups and the England and Wales-born population is also of aetiological interest.

In this paper we analyse site-specific cancer mortality in 1970-85 in West African-, East African-, and Caribbean-born immigrants, and compre this with cancer mortality in the England and Wales-born.

### Methods

The immigrants were divided into East African-, West African- and Caribbean-born as detailed in Table I. Data on cancer deaths in 1970-85 in residents of England and Wales, by country of birth, were obtained from the Office of Population Censuses and Surveys. Deaths were coded to ICD8 in 1970-8 and to ICD9 in 1979-85. Deaths occurring in 1970-8 were bridge coded to ICD9 using the categories in Tables IIa and IIb. The numbers of deaths due to site-specific cancers formed the numerator for calculation of rates in each of the immigrants groups. The denominator was the 1971 census population by country of birth for deaths in 1970-3, the 1981 census population by country of birth for 1979-85, and the mean of these two census populations for 1974-8. For East Africans, the 1981 population was used for the 1974-8 period as most immigration from this area occurred in 1972-73 (OPCS, 1981).

Relative risks were calculated by Poisson regression (Kaldor *et al.*, 1990). Age-adjustment was performed using age groups 0-14, 15-19, 20-24, . . ., 80-84, 85 +. Relative risks were calculated separately for each country within these regions, but grouped into Commonwealth East and West Africa and Commonwealth Caribbean for presentation.

### *Period since migration*

Year of entry into England and Wales is not recorded at death certification. Calendar period is a reasonable proxy for time since migration for Caribbean immigrants, because there has been little immigration from the Caribbean since the early 1960's (Watson, 1977) but not for other immigrant groups. Analyses for the effect of calendar period on cancer mortality risks were therefore undertaken only for Caribbean immigrants. Relative risks were calculated for 1970-3, 1974-8, and 1979-82, using denominators as above. A Poisson model was fitted with a term for period and region of birth, and compared with one which contained additionally a country of birth-period interaction. In this way, change in mortality rates over time was significant only if the pattern deviated from that for the England and Wales-born.

### *Social class*

The effect of social class on mortality rates was also examined through Poisson regression. Numerator data were from social class at death, derived from the occupation as recorded on death certificates. This was 100% coded only in

Correspondence: A.E. Grulich, Department of Epidemiology and Population Sciences, London School of Hygiene and Tropical Medicine, Keppel Street, London WC1E 7HT, UK.
Received 27 February 1992; and in revised form 26 June 1992.

**Table I** African and Caribbean born resident populations of England and Wales, at 1981 census, by age and sex

| Age groups (years) | West African born[a] Males No. | % | Females No. | % | East African born[b] Males No. | % | Females No. | % | Caribbean born[c] Males No. | % | Females No. | % |
|---|---|---|---|---|---|---|---|---|---|---|---|---|
| 0–14 | 2,341 | 8.3 | 2,443 | 10.7 | 12,865 | 12.7 | 12,493 | 13.6 | 2,860 | 2.0 | 2,800 | 1.9 |
| 15–24 | 5,932 | 21.1 | 6,506 | 28.6 | 35,447 | 35.0 | 33,742 | 36.8 | 18,161 | 12.6 | 21,591 | 14.4 |
| 25–34 | 9,384 | 33.4 | 7,919 | 34.8 | 32,063 | 31.7 | 27,198 | 29.7 | 23,341 | 16.2 | 32,958 | 22.0 |
| 35–44 | 6,261 | 22.3 | 4,389 | 19.3 | 12,374 | 12.2 | 10,701 | 11.7 | 34,859 | 24.3 | 39,984 | 26.7 |
| 45–54 | 2,932 | 10.4 | 1,095 | 4.8 | 6,219 | 6.1 | 5,440 | 17.6 | 39,803 | 27.7 | 33,615 | 22.4 |
| 55–64 | 951 | 3.4 | 245 | 1.1 | 1,747 | 1.7 | 1,477 | 1.6 | 19,147 | 13.3 | 13,332 | 8.9 |
| 65+ | 316 | 1.1 | 131 | 0.6 | 485 | 0.5 | 538 | 0.6 | 5,538 | 3.9 | 5,643 | 3.8 |
| Total | 28,117 | 100 | 22,728 | 100 | 101,200 | 100 | 91,589 | 100 | 143,709 | 100 | 149,923 | 100 |

[a]Sierra Leone, Ghana, Nigeria, Gambia; [b]Kenya, Malawi, Tanzania, Uganda, Zambia; [c]Barbados, Jamaica, Trinidad and Tobago, West Indies associated states, West Indies (so stated), Belize, Guyana, other Commonwealth Caribbean.

**Table IIa** Risks of cancer mortality in immigrant groups compared to England and Wales natives, 1970–1985: males

| ICD 9 code and site | West African immigrants No. | RR (95% C.I.) | East African immigrants No. | RR (95% C.I.) | Caribbean immigrants No. | RR (95% C.I.) |
|---|---|---|---|---|---|---|
| 141, 143–5 Oral cavity | 1 | 0.6 (0.1–4.3) | 14 | 4.3 (2.6–7.3) | 12 | 0.5 (0.3–1.0) |
| 146, 148–9 Pharynx | 1 | 0.9 (0.1–6.0) | 6 | 2.6 (1.6–5.7) | 12 | 0.7 (0.4–1.2) |
| 147 Nasopharynx | 2 | 3.7 (0.9–15.0) | 1 | 0.8 (0.1–5.5) | 19 | 3.1 (2.0–4.9) |
| 150 Oesophagus | 18 | 2.5 (1.6–4.0) | 9 | 0.7 (0.3–1.3) | 81 | 0.8 (0.6–0.9) |
| 151 Stomach | 25 | 1.2 (0.8–1.8) | 12 | 0.4 (0.2–0.6) | 339 | 1.1 (1.0–1.2) |
| 153–4 Colon and rectum | 26 | 1.0 (0.7–1.5) | 35 | 0.7 (0.5–1.0) | 189 | 0.5 (0.5–0.6) |
| 155.0–155.1 Liver, stated primary | 50 | 31.6 (23.8–41.9) | 4 | 1.1 (0.4–3.0) | 114 | 5.3 (4.4–6.3) |
| 156 Gallbladder | 2 | 1.5 (0.4–6.1) | 1 | 0.4 (0.1–2.8) | 18 | 1.0 (0.6–1.5) |
| 157 Pancreas | 19 | 1.8 (1.1–3.0) | 7 | 0.4 (0.2–0.8) | 131 | 0.9 (0.7–1.0) |
| 161 Larynx | 3 | 1.5 (0.5–4.7) | 3 | 0.8 (0.3–2.4) | 10 | 0.3 (0.2–0.6) |
| 162 Lung | 60 | 0.7 (0.6–0.9) | 51 | 0.3 (0.2–0.4) | 473 | 0.4 (0.3–0.4) |
| 172 Melanoma | 2 | 0.5 (0.1–1.9) | 2 | 0.2 (0.1–1.0) | 11 | 0.3 (0.2–0.6) |
| 185 Prostate | 26 | 3.5 (2.4–5.1) | 7 | 0.6 (0.3–1.2) | 201 | 1.7 (1.5–2.0) |
| 186 Testis | 1 | 0.2 (0.02–1.2) | 3 | 0.2 (0.1–0.5) | 4 | 0.1 (0.1–0.4) |
| 187 Penis | 1 | 2.1 (0.3–14.8) | 2 | 2.6 (0.5–8.3) | 9 | 1.5 (0.9–2.9) |
| 188 Bladder | 3 | 0.4 (0.2–1.3) | 8 | 0.6 (0.3–1.3) | 61 | 0.6 (0.4–0.7) |
| 189 Kidney | 6 | 1.2 (0.5–2.5) | 7 | 0.6 (0.3–1.3) | 42 | 0.6 (0.4–0.8) |
| 191–2 Brain and nervous system | 6 | 0.5 (0.2–1.0) | 32 | 1.0 (0.7–1.4) | 62 | 0.5 (0.4–0.6) |
| 155.2, 159, 165, 195–9 Ill-defined and unspecified | 23 | 2.1 (1.4–3.1) | 14 | 0.6 (0.4–1.0) | 153 | 1.0 (0.8–1.1) |
| 200, 202 Non-Hodgkin's lymphoma | 20 | 2.6 (1.7–4.0) | 13 | 0.7 (0.4–1.2) | 117 | 1.6 (1.3–1.9) |
| 201 Hodgkin's disease | 4 | 0.6 (0.3–1.6) | 6 | 0.3 (0.2–0.8) | 37 | 0.9 (0.7–1.2) |
| 203 Multiple myeloma | 10 | 4.1 (2.2–7.6) | 9 | 1.9 (1.0–3.7) | 80 | 2.2 (1.7–2.7) |
| 204–8 Leukaemia | 18 | 1.4 (0.9–2.3) | 53 | 1.4 (1.1–1.9) | 114 | 1.1 (0.9–1.3) |
| 140–208 All cancer | 343 | 1.38 (1.25–1.54) | 326 | 0.63 (0.56–0.70) | 2388 | 0.71 (0.68–0.74) |

**Table IIb** Risks of cancer mortality in immigrant groups compared to England and Wales natives, 1970–1985: females

| ICD 9 code and site | West African immigrants No. | RR (95% C.I.) | East African immigrants No. | RR (95% C.I.) | Caribbean immigrants No. | RR (95% C.I.) |
|---|---|---|---|---|---|---|
| 141, 143–5 Oral cavity | 3 | 9.2 (3.0–28.3) | 14 | 10.7 (6.3–18.2) | 9 | 1.1 (0.6–2.0) |
| 146, 148–9 Pharynx | 0 | – | 1 | 0.7 (0.1–4.7) | 3 | 0.3 (0.1–0.9) |
| 147 Nasopharynx | 1 | 6.2 (0.9–43.0) | 1 | 1.6 (0.2–10.8) | 4 | 1.6 (0.6–4.3) |
| 150 Oesophagus | 1 | 0.8 (0.1–5.5) | 14 | 2.4 (1.4–4.1) | 27 | 0.6 (0.4–0.9) |
| 151 Stomach | 6 | 1.5 (0.7–3.4) | 12 | 0.7 (0.4–1.3) | 129 | 1.0 (0.9–1.2) |
| 153–4 Colon and rectum | 10 | 1.2 (0.6–2.2) | 19 | 0.5 (0.3–0.8) | 136 | 0.5 (0.4–0.6) |
| 155.0–155.1 Liver, stated primary | 3 | 5.4 (1.7–16.7) | 4 | 1.8 (0.7–4.8) | 34 | 3.2 (2.2–4.4) |
| 156 Gallbladder | 2 | 3.7 (0.9–14.8) | 2 | 0.8 (0.2–3.3) | 38 | 2.0 (1.5–2.8) |
| 157 Pancreas | 4 | 1.7 (0.6–4.5) | 9 | 0.8 (0.4–1.6) | 59 | 0.7 (0.6–0.9) |
| 161 Larynx | 0 | – | 1 | 1.2 (0.2–8.3) | 1 | 0.2 (0.0–1.2) |
| 162 Lung | 2 | 0.2 (0.1–0.9) | 25 | 0.6 (0.4–0.9) | 100 | 0.3 (0.3–0.4) |
| 172 Melanoma | 3 | 1.2 (0.4–3.6) | 1 | 0.1 (0.02–0.8) | 8 | 0.2 (0.1–0.5) |
| 174 Breast | 37 | 1.3 (1.0–1.8) | 102 | 0.9 (0.7–1.1) | 538 | 0.8 (0.7–0.9) |
| 179, 182 Uterus (corpus and unspecified) | 3 | 2.0 (0.7–6.2) | 4 | 0.6 (0.2–1.5) | 68 | 1.3 (1.0–1.6) |
| 180 Cervix | 5 | 0.6 (0.3–1.4) | 12 | 0.4 (0.2–0.7) | 185 | 1.3 (1.1–1.5) |
| 181 Placenta | 0 | – | 0 | – | 5 | 7.1 (2.8–17.6) |
| 183 Ovary | 5 | 0.6 (0.3–1.5) | 18 | 0.5 (0.3–0.8) | 93 | 0.5 (0.4–0.6) |
| 188 Bladder | 0 | – | 3 | 0.8 (0.2–2.4) | 16 | 0.5 (0.3–0.8) |
| 189 Kidney | 2 | 1.6 (0.4–6.2) | 4 | 0.8 (0.3–2.0) | 21 | 0.7 (0.5–1.1) |
| 191–2 Brain and nervous system | 1 | 0.2 (0.0–1.2) | 14 | 0.7 (0.4–1.2) | 26 | 0.4 (0.2–0.5) |
| 155.2, 159, 165, 195–9 Ill-defined and unspecified | 6 | 1.6 (0.7–3.4) | 9 | 0.5 (0.3–1.1) | 97 | 0.9 (0.7–1.1) |
| 200, 202 Non-Hodgkin's lymphoma | 3 | 1.1 (0.4–3.5) | 11 | 1.1 (0.6–2.0) | 92 | 2.1 (1.7–2.6) |
| 201 Hodgkin's disease | 2 | 0.8 (0.2–3.2) | 3 | 0.4 (0.1–1.1) | 18 | 0.9 (0.5–1.4) |
| 203 Multiple myeloma | 1 | 1.6 (0.2–11.5) | 5 | 1.7 (0.7–4.1) | 46 | 2.0 (1.5–2.7) |
| 204–8 Leukaemia | 11 | 1.8 (1.0–3.2) | 32 | 1.3 (1.0–1.9) | 75 | 1.1 (0.9–1.3) |
| 140–208 All cancer | 119 | 1.14 (0.96–1.37) | 341 | 0.80 (0.72–0.89) | 1890 | 0.76 (0.73–0.80) |

1970–73, 1979–81, and 1983–85, and was reasonably complete only at working ages. Thus complete data on social class at death were available only for those aged 15–64 who died in these years. Denominator data were available from the 1971 census. In the 1981 census, however, social class by country of birth was not tabulated, but was available for 1% of the census population from the Longitudinal Study, a 1% sample of the population of England and Wales. For Caribbean immigrants these 1981 results were minimally different from the 1971 data. For East and West African immigrants, the numbers in each stratum of social class by age by sex were too small to given reliable estimates of the social class distribution in 1981. Consequently, social class adjustment was performed for Caribbean but not African immigrants. The age-specific proportion of Caribbean immigrants in each social class from the Longitudinal Study were applied to the population by country of birth at the 1981 census to estimate the population at risk in 1979–81 and 1983–85.

The limitations of numerator availability meant that for most sites, only 20–40% of cancer deaths in Caribbean immigrants could be included in the social class analyses. The relatively small numbers of cancer deaths remaining for analysis meant that the power to detect an effect of social class on cancer mortality was limited, and analysis had to be restricted to the most common sites.

### **Results** (Tables IIa and IIb)

Over 5,000 deaths from cancer were registered in the immigrant groups studied during 1970–85. The highest overall cancer death rates occurred in West African males, whose rates were nearly 40% higher than in the England and Wales-born. Caribbean and East African male immigrants had significantly low all cancer rates. The deviation of all cancer risk from unity was greater in males than in females in each migrant group.

### *Oro-pharyngeal cancer*

East African immigrants had mortality from cancers of the oral cavity about five to ten times England and Wales native rates and also had significantly raised mortality from cancers of the pharynx (males only). West African females also had significantly raised oral cancer mortality. Nasopharyngeal cancer mortality was significantly raised in Caribbean male immigrants.

### *Digestive system cancer*

Oesophageal cancer mortality was significantly raised in West African male immigrants and East African female immigrants, but mortality rates were significantly low in Caribbean immigrants. Stomach cancer mortality was significantly low in East African male immigrants. Colorectal cancer mortality in Caribbean immigrants was about half that of the England and Wales-born, and was also significantly low in East African immigrants.

Liver cancer mortality showed the most variation of any cancer. Rates in East African immigrants were raised non-significantly. Caribbean immigrants had significantly high rates, three to five times those in the English-born. Mortality in West African male immigrants was over 30 times the natives' rates and it was also raised in West African female immigrants. Gallbladder cancer mortality was significantly raised in Caribbean female immigrants. Pancreatic cancer mortality was significantly raised in males from West Africa, but in the other immigrant groups was signifiantly less common than in the England and Wales-born.

### *Respiratory cancer*

Lung cancer mortality rates were significantly low in all immigrant groups. Rates in West African born males were closest to the rate of the England and Wales-born.

### *Reproductive and urinary cancer*

Breast cancer mortality in Caribbean immigrants was significantly lower than in England and Wales natives. Cervical and uterine cancer mortality was significantly raised in Caribbean immigrants but in immigrants from East Africa mortality from cervical cancer was significantly low. Ovarian cancer mortality was significantly low in East African and Caribbean immigrants. Placental cancer mortality was significantly raised in Caribbean immigrants.

Prostatic cancer mortality was raised in West African and Caribbean immigrants, but not in men from East Africa. Testicular cancer mortality was low and penile cancer mortality non-significantly raised in all immigrant groups. Bladder cancer mortality was significantly low in Caribbean immigrants of both sexes, and kidney cancer mortality significantly low in Caribbean male immigrants.

### *Haematological cancer*

Mortality from non-Hodgkin's lymphoma was significantly raised in male West African and all Caribbean immigrants, but not in East African immigrants. Mortality from Hodgkin's disease was slightly low in all immigrant groups, but only in East African males was this statistically significant. Multiple myeloma mortality was significantly high in all male immigrants, as well as female Caribbean immigrants. It was non-significantly raised in the other female immigrants. Leukaemia mortality was significantly raised in East African immigrants and West African females but not in Caribbean immigrants.

### *Other cancers*

Caribbean and East African immigrants had significantly low mortality from malignant melanoma. West African and Caribbean immigrants each had low mortality from brain and nervous system cancer, but only in the latter was this significant.

### *Time trends*

Significantly decreasing trends, convergent towards England and Wales rates, were present for liver cancer in males and females, and non-Hodgkin's lymphoma in females from the Caribbean (Table III). There were no cancer sites for which the Caribbean-born had trends significantly divergent from those in England and Wales.

### *Social class adjustment*

East and West African immigrants had a higher social class distribution than the England and Wales-born, whereas Caribbean immigrants were concentrated in the manual classes (Table IV). Analysis of the effect of social class on cancer risk was possible only on a subset of the Caribbean-born subjects (see Methods), whose age-adjusted risks could be different from those for all Caribbean immigrants in Tables IIa and b. For example, only 20% of deaths from cervical cancer in Caribbean-born women could be included in this analysis, and the RR for cervical cancer in Caribbean-born women eligible for the analysis was 1.8 (95% CI 1.4–2.4), compared to 1.3 (1.1–1.5) for all Caribbean-born women. After adjustment for social class, the RR in the eligible subgroup decreased to 1.6 (1.2–2.1). Risks for stomach and lung cancer, which are known to have strong social class gradients, were scarcely affected by social class adjustment.

### **Discussion**

It is desirable in migrant studies to compare rates of disease in migrants to rates in both their new country and their country of origin. This is difficult when discussing migrants from developing countries, where there are few data. While

**Table III** Cancer sites with significant divergence in mortality from England and Wales-born rates over time in Caribbean immigrants, 1970–83[a]

| | *1970–3 RR*[b] | *1974–8 RR*[b] | *1979–83 RR*[b] | *$\chi_2^2$ for region of birth-period interaction* | *$\chi_1^2$ for difference in linear trend*[c] |
|---|---|---|---|---|---|
| Liver: males | | | | | |
| E and W | 1.0 | 1.1 | 1.8 | 5.99 | 5.50* |
| Caribbean | 7.3 | 5.8 | 6.6 | | |
| Liver: females | | | | | |
| E and W | 1.0 | 1.1 | 2.2 | 12.28** | 10.93** |
| Caribbean | 5.4 | 4.5 | 2.1 | | |
| Non-Hodgkin's lymphoma: females | | | | | |
| E and W | 1.0 | 1.1 | 1.4 | 7.13* | 6.24* |
| Caribbean | 2.9 | 2.9 | 2.0 | | |

[a]Discrepancies from the overall relative risks in Tables IIa and IIb are because of the exclusion from this table of deaths in 1984–5. [b]Relative risks (England and Wales rates in 1970–3 = 1) estimated from a model including age, time period, region of birth, and region of birth × time period interaction, with time period included as a categorical variable. [c]Test for significance of region of birth x period interaction with time period included as a linear variable. *$P<0.05$; **$P<0.01$.

**Table IV** Social class distribution of England and Wales-born and immigrant groups, by sex, in England and Wales at 1971 census (percentages)

| *Region of birth* | *Social class 1* | *2* | *3N* | *3M* | *4* | *5* | *Total* |
|---|---|---|---|---|---|---|---|
| *Males* | | | | | | | |
| England and Wales | 4.8 | 18.5 | 12.3 | 38.8 | 17.6 | 8.0 | 100 |
| Caribbean C'wealth | 1.3 | 4.4 | 4.6 | 46.3 | 26.7 | 16.8 | 100 |
| West Africa | 10.6 | 15.1 | 25.2 | 21.2 | 20.2 | 7.8 | 100 |
| East Africa | 10.9 | 15.9 | 18.6 | 27.8 | 22.0 | 4.8 | 100 |
| *Females* | | | | | | | |
| England and Wales | 0.9 | 16.7 | 38.9 | 10.4 | 25.6 | 7.5 | 100 |
| Caribbean C'wealth | 0.3 | 29.8 | 13.4 | 9.6 | 39.8 | 7.1 | 100 |
| West Africa | 1.1 | 33.7 | 27.0 | 11.5 | 22.4 | 4.3 | 100 |
| East Africa | 2.0 | 23.1 | 35.4 | 10.9 | 27.0 | 1.5 | 100 |

rates of cancer mortality are not known reliably, a good deal is known about the relative frequencies of cancer in Africa and the Caribbean.

Many of the most common cancers in Africa are believed to be viral in origin. These include liver cancer, which is particularly common in West Africa, cervical cancer, and non-Hodgkin's lymphoma (Parkin *et al.*, 1988). In East Africa, Kaposi's sarcoma and penile cancer, also believed to be infective in origin, are common. The other most common cancers, with regional variation within Africa, are cancers of the oesophagus, stomach, prostate, bladder and breast (Waterhouse *et al.*, 1982; Bayo *et al.*, 1990; Bah *et al.*, 1990). There appear to be no data on cancer occurrence in the Asian-origin population of East Africa.

The major causes of cancer mortality in the Caribbean are similar to those in Africa. Cancers with high risks by international standards include cancers of the prostate, cervix, liver, stomach and penis (Hamilton & Persaud, 1981; Persuad, 1976; 1986). Cancers common in Western societies, such as colon, lung and breast cancer, are more common in the Caribbean than in Africa but not so frequent as in England and Wales (Waterhouse *et al.*, 1982).

In comparing the cancer rates in migrants to the cancer patterns in their home countries, it is important to recognise that the migrant groups dealt with in this paper have undergone considerable selection processes. About 75% of East African immigrants are of Asian ethnicity, 13% are white, and only 6% are of African ethnicity (OPCS monitor, 1983). A larger proportion are of social classes 1 and 2 than are of these classes in the native population of England and Wales (Table IV). Although no information on ethnicity is collected specifically for West Africans it is known that few persons of Asian origin immigrated from West Africa, and that they are predominantly ethnic Africans. Many West African immigrants are mature students, and many are of upper socioeconomic class (Watson, 1977). Caribbean immigrants are of similar racial origin to the West African immigrants, and 92% classify themselves as ethnically African or West Indian (OPCS Monitor, 1983). A few, mainly from Trinidad, are Asian. In England and Wales Caribbean male immigrants work predominantly in manual occupations (Table IV). The African- and Caribbean-born populations in England and Wales include relatively few persons of childhood and elderly ages compared to the England and Wales-born population, reflecting the dates of the main migrations.

To consider the possible effects of environment and genetics on cancer risk, we discuss here three comparisons amongst these migrant groupings. The first is between the two predominantly ethnic African immigrant groups, i.e. the West African immigrants, who are of comparatively high social class in England and Wales, and the Caribbean immigrants, who are of lower social class. The second comparison is between the two groups exposed to an African environment, the ethnic African West Africans and the predominantly ethnic Asian East Africans. Finally, there were cancers which showed the same mortality pattern in all these groups of immigrants.

### (1) *Comparison of West African and Caribbean immigrants*

### *Cancers of high mortality in both West African and Caribbean immigrants*

*(a) Prostatic cancer* The highest rates of this cancer worldwide are in US blacks (McKay *et al.*, 1982), and rates are also higher in ethnic Africans than in whites in Brazil (Bouchardy *et al.*, 1991). Age-standardised incidence rates in Jamaica are about 1.5 times those in England and Wales (Waterhouse *et al.*, 1982). In West Africa the incidence has not been thought to be high (Waterhouse *et al.*, 1982; Parkin *et al.*, 1988) but this may be related to a lack of diagnostic facilities. In the present data rates were high in both West African and Caribbean immigrants, but not in the predominantly ethnic Asian East African immigrants. This is compatible with a genetic factor predisposing ethnic Africans to prostate cancer. The nature of the factor is unclear. Young black men in the US have been found to have higher levels of serum testosterone, the main hormone promoting growth of epithelial tissue in the prostate, than in whites, and it has been postulated that this may predispose to prostatic cancer (Ross *et al.*, 1986). Higher levels of testosterone have also been found in patients with prostatic cancer than in controls (Jackson *et al.*, 1981). Studies of immigrants from countries with low incidence rates of prostatic cancer to the US have shown that rates of prostatic cancer increase towards the US levels (Jackson *et al.*, 1981), so environmental factors are likely to be important. We found that rates of prostatic cancer in Caribbean males did converge towards the rates in England and Wales with calendar time (a proxy for time since migration), but not significantly so.

*(b) Liver cancer* The marked variation in mortality from this cancer in the immigrant groups probably mainly reflects levels of infection with hepatitis B virus. Mortality rates from viral hepatitis (ICD 9 070), which includes hepatitis A, B, and other and unspecified viral hepatitis, but is likely as a cause of death to be mainly due to hepatitis B, showed a similar pattern in African and Caribbean immigrants to that of liver cancer. In West African immigrants, the RR of mortality from viral hepatitis was 23.7 (13.9–40.4) in males and 13.2 (5.5–32.0) in females. In East African immigrants it was 1.7 (0.6–5.4) in males and 1.3 (0.3–5.4) in females, and in Caribbean immigrants it was 1.5 (0.7–3.1) in males and 4.3 (2.7–7.0) in females (Grulich *et al.*, unpublished data). These high risks were due to excess deaths from hepatitis in adults, but not in children. High rates of chronic infection with hepatitis B have been well documented in West Africans in West Africa (Anthony, 1984) and around 90% of populations in this area have evidence of past or present infection with hepatitis B (Coursaget *et al.*, 1984). Given that transmission of hepatitis B in West Africa is predominantly horizontal, in young children (Hall *et al.*, 1991), our finding of very high rates of liver cancer mortality in West African male immigrants, but only slightly high rates in females, is somewhat surprising, but a similar sex ratio for incidence of liver cancer has been found in West Africa (Bah *et al.*, 1990; Bayo *et al.*, 1990). This may indicate that co-factors such as alcohol consumption, exposure to aflatoxin, or infection with other hepatitis viruses may be important. Possible indirect support for the role of alcohol is that rates of oesophageal cancer mortality were also raised in West African male immigrants. The convergence of rates of liver cancer towards the England and Wales rates in Caribbean immigrants also suggests the action of co-factors, although the trend could also in theory be related to cohort-based trends in liver cancer mortality originating from Caribbean exposures.

Liver cancer is a major public health problem in the West African community in England and Wales. It caused 15% of all cancer deaths in the period of this study in West African male immigrants. The mechanism of transmission of hepatitis B in young children in West Africa is unclear, but may be related to the presence of biting insects and exudative scars (Vall Mayans *et al.*, 1990). The transmission dynamics of hepatitis B have not been studied in the West African-born population in England and Wales, but evidence from other industrialised countries suggests that horizontal transmission within families may occur (Christenson, 1986). The possibility of horizontal transmission within the West African immigrant community is also suggested by our finding of raised risks of death from viral hepatitis in adult West African immigrants, although some of these cases may be due to incorrect coding of deaths due to chronic active hepatitis following childhood infection. In the US, high rates of liver cancer have been maintained by immigrants from high risk areas for at least the first and second generations, although these data are from Chinese immigrants in whom vertical transmission of hepatitis B predominates (Anthony, 1984). The magnitude of the problem in England suggests that investigation of hepatitis B serology in the West African community should be an urgent task. The current official recommendation that all non-Caucasian ethnic groups should be screened for hepatitis B during pregnancy, and the baby immunized only if the mother is seropositive (D.O.H., 1990) may not be sufficient to curtail transmission of hepatitis B if children mix within their own ethnic group with non-screened children.

*Cancers of low mortality in both West African and Caribbean immigrants*

*Brain/Nervous system* Mortality rates from these cancers were around half the England and Wales natives' rates in both West African and Caribbean immigrants, but were not low in East African immigrants. Mortality rates from these cancers in US blacks are also about 50–60% of rates in whites (Schoenberg, 1982; McKay *et al.*, 1982). Brain cancer has been linked to high socio-economic status in the US (Brownson *et al.*, 1990), and in England and Wales (Davey-Smith *et al.*, 1991). The occurrence of low nervous system cancer mortality risks in both Caribbean and West African immigrants makes social class related factors an unlikely explanation of the racial differences found in this study, although the rates in West Africans were only of borderline significance. The reason for low rates of brain cancer in blacks is unclear.

(2) *Comparison of East and West African immigrants*

*Cancers of high mortality in both East and West African immigrants*

*(a) Leukaemia* Raised mortality rates from leukaemia were present in African immigrants and were not confined to any cell type. Mortality from leukaemia is less common in blacks than whites in the US (McKay *et al.*, 1982), and is thought to be uncommon in Africa (Parkin *et al.*, 1988) although this apparently low risk may be due to lack of diagnostic facilities.

*(b) Oral cancer* Rates of oral cancer were raised in East African immigrants and in West African-born females. The high rates in East Africans may be related to the Asian ethnicity of most of these immigrants. Betel-chewing, which has been shown to increase the risk of oral cancer (Mahboubi & Sayed, 1982), is common amongst ethnic Asians but not ethnic Africans. This cannot, however, explain the raised risk in West African females.

*Cancers of low mortality in both East and West African immigrants*

*Cervical cancer* Given that cervical cancer is proportionately the most common cancer in women in much of sub-Saharan Africa (Parkin, 1986), it was surprising to find that the East African immigrants had a low risk of this cancer and that West Africans, based on few cases, had a non-significantly lowered risk. Risks in immigrants from the Indian subcontinent to England and Wales are not decreased (Grulich *et al.*, unpublished data). The low rates of this cancer in both of these groups may be related to their relatively high social class, as cervical cancer mortality is known to be strongly related to social class. A similar picture exists in the US, where the high rates in blacks are not maintained when rates are adjusted for social class (Christopherson & Nealon, 1981). There were insufficient cases of cervical cancer in Africans in our data to assess the impact of social class adjustment.

(3) *Comparison of all immigrant groups*

*Cancers of high mortality in all immigrants*

*Multiple myeloma* Raised rates have been described in blacks compared to whites in the US (National Cancer Institute, 1986) and Brazil (Bouchardy *et al.*, 1991), and there are high rates in blacks in Jamaica (Blattner, 1982). Despite the high rates in East African immigrants, who are largely of Indian origin, risks were not raised in immigrants from the Indian subcontinent. The RR was 1.0 (0.8–1.2) in males and 0.9 (0.7–1.2) in females born in the Indian subcontinent (Grulich *et al.*, unpublished data). This would be compatible with an environmental agent in Africa predisposing to multiple myeloma. The fact that rates have now been found high in black populations in three continents, including blacks in the US who are separated by several generations from Africa, however, may be indicative of a genetic predisposition.

*Cancers of low mortality in all immigrants*

*(a) Testicular cancer* Relative risks are also low for this cancer in US blacks (National Cancer Institute, 1986), and in Indian immigrants to England and Wales (Grulich *et al.*, unpublished data). There is a strong tendency for testicular cancer to occur in upper socio-economic classes, and it has been postulated that this may explain much of the racial difference in US rates (Schottenfeld & Warshauer, 1982). Our finding of low rates even in West African immigrants, whose social class tends to be high, tends to point against this. The only substantial accepted risk factor for testicular cancer is cryptorchidism. There are no known racially determined risk factors.

*(b) Ovarian cancer* showed a similar pattern in migrant groups to cancer of the testis. Aetiological factors for this tumour include low fertility, and thus the high fertility rates of these immigrants may explain some of the racial differences. In 1971, total fertility rates were 2.3 in England and Wales-born women compared to 2.9 in African-born women and 3.4 in West Indian-born women (Immigrant Statistics Unit, 1978). Mortality from breast cancer, which has some similar risk factors to ovarian cancer, was also low in Caribbean immigrants, but was high in West African immigrants.

*(c) Lung cancer* In Caribbean immigrants, lung cancer rates were about one third of the rates in England and Wales, and there was no increasing trend. Rates in East African immigrants were slightly higher and in West African male immigrants, the relative risk 0.72 was the highest of all the immigrant groups. These differences are likely to reflect differences in smoking patterns.

*(d) Melanoma* The low rates of this tumour in non-white populations are well recognised (Crombie, 1979). Rates were very low in East African and Caribbean immigrants, but in West African immigrants were non-significantly low in males and close to 1 in females, based on very few cases.

*All-cancer rates by region of birth*

All-cancer rates were low in both Caribbean and East African immigrants. The main cause of this low mortality was the low rates of cancers which are associated with Western lifestyle, particularly lung and colorectal cancers. Relative risks were particularly low in men, which reflects the larger influence that tobacco-related tumours have on cancer rates in males than in females. Many of the cancers with raised mortality in these migrants, such as nasopharyngeal cancer, liver cancer, cervical cancer, non-Hodgkin's Lymphoma, multiple myeloma and leukaemia are those in which an infective cause has been postulated and the raised mortality from oral cancer in East African immigrants may be related to betel chewing. The reasons for the raised mortality from oesophageal mortality in East African-born females and from prostate and placental cancers in Caribbeans are largely unclear.

The finding of high all-cancer mortality rates in West African immigrants is at odds with the low all-cancer incidence rates which have been reported from West Africa (Waterhouse *et al.*, 1982). At least part of the reason for the discrepancy is likely to be under-reporting of cancer in African registries. It is possible that a few cases may have travelled to the UK for treatment, but this would not explain the site distribution. These migrants are a selected group whose cancer rates may not necessarily reflect that of their home countries. Migrants from West Africa tend to come from the upper strata of West African society, in which alcohol consumption is widespread, and cigarette smoking is probably more common than in other African migrants. These people are in a state of transition from a traditional African lifestyle to a more Westernised one. The present data show that they still get the cancers associated with West Africa, such as liver cancer, but their rates of cancers usually associated with Western countries such as colon, breast and lung cancer are also higher than the other immigrant groups discussed here.

We are grateful to the Cancer Research Campaign for funding the study. We thank the Office of Population Censuses and Surveys for the provision of mortality data, and the Social Science Research Unit, City University, for data on social class by country of birth from the Longitudinal Study.

**References**

ANTHONY, P.P. (1984). Hepatocellular carcinoma: an overview. In *Virus Associated Cancer in Africa*. Williams, O., O'Conor, G., De-The, G. & Johnson, C. (eds.). pp. 3–29. IARC Scientific publication no. 63. IARC: Lyon.

BAH, E., HALL, A.J. & INSKIP, H.M. (1990). The first two years of the Gambian cancer registry. *Br. J. Cancer*, **62**, 647–650.

BAYO, S., PARKIN, D.M., KOUMARÉ, A.K., DIALLO, A.N., BA, T., SOUMARÉ, S. & SANGARE, S. (1990). Cancer in Mali, 1987–88. *Int. J. Cancer*, **45**, 679–684.

BLATTNER, W.A. (1982). Multiple myeloma and macroglobulinaemia. In: *Cancer Epidemiology and Prevention*. Schottenfeld, D. & Fraumeni, J.F. Jr. (eds.) pp. 795–813. WB Saunders: Philadelphia.

BOUCHARDY, C., MIRRA, A.P., KHLAT, M., PARKIN, D.M., DE SOUZA, J.M. & GOTLEIB, S.L. (1991). Ethnicity and cancer risk in Sao Paulo, Brazil. *Cancer Epidemiol. Biomarkers and Prevention*, **1**, 21–27.

BROWNSON, R.C., REIF, J.S., CHANG, J.C. & DAVIS, J.R. (1990). An analysis of occupational risks for brain cancer. *Am. J. Public Health*, **80**, 169–172.

CHRISTENSON, B. (1986). Epidemiological aspects of the transmission of hepatitis B by HBsAg-positive children. *Scan. J. Infect. Dis.*, **18**, 105–109.

CHRISTOPHERSON, W.M. & NEALON, N.A. (1981). Uterine cancer: a comparative study of black and white women. In *Cancer Among Black Populations*. Mettlin, C. & Murphy, G. (eds.). pp. 185–195. Alan R. Liss: New York.

COURSAGET, P., CHIRON, J.P., BARRES, J.L., BARIN, F., COTTEY, P., TORTEY, E., YVONNET, B., DIOP, B., MBOUP, S., DIOP-MAR, I., KOCHELEFF, P., PERRIN, J. & DUFLO, B. (1984). Hepatitis B serological markers in Africans with liver cirrhosis and hepatocellular carcinoma. In *Virus Associated Cancer in Africa*. Williams, O., O'Conor, G., De-The, G. & Johnson, C. (eds.) pp. 181–198. IARC scientific publication no. 63. IARC: Lyon.

CROMBIE, I.K. (1979). Racial differences in melanoma incidence. *Br. J. Cancer*, **40**, 185–193.

DAVEY-SMITH, G., LEON, D., SHIPLEY, M.J. & ROSE, G. (1991). Socioeconomic differentials in cancer among men. *Int. J. Epidemiol.*, **20**, 339–345.

DEPARTMENT OF HEALTH, WELSH OFFICE, SCOTTISH HOME AND HEALTH DEPARTMENT (1990). *Immunisation against infectious diseases*. HMSO: London.

HALL, A.J. & SMITH, P.G. (1991). Hepatitis B control in African countries. *Lancet*, **337**, 247.

HAMILTON, P.J. & PERSUAD, V. (1981). Cancer among blacks in the West Indies. In *Cancer Among Black Populations*, Mettlin, C. & Murphy, G. (eds.) pp. 1–15. Alan R. Liss: New York.

HOLMES, C. (1988). *John Bull's Island, Immigration and British Society, 1871–1971*. Macmillan Education: London.

IMMIGRANT STATISTICS UNIT, OPCS (1978). Marriage and birth patterns among the New Commonwealth and Pakistani population. *Pop. Trends*, **11**, 5–9.

JACKSON, M.A., KOVI, J., HESHMET, M.Y., JONES, G.W., RAO, M.S. & AHLUWALIA, B.S. (1981). Factors involved in the high incidence of prostatic cancer among American blacks. In *Cancer Among Black Populations*. Mettlin, C. & Murphy, G. (eds) pp. 111–132. Alan R. Liss: New York.

KALDOR, J., KHLAT, M., PARKIN, D.M., SHIBOSKI, S. & STEINITZ, R. (1990). Log-linear models for cancer risk among migrants. *Int. J. Epidemiol.*, **19**, 233–239.

MAHBOUBI, E. & SAYED, G.M. (1982). Oral cavity and pharynx. In *Cancer Epidemiology and Prevention*. Schottenfeld, D. & Fraumeni, J.F. Jr. (eds). pp. 583–596. WB Saunders: Philadelphia.

MARMOT, M.G., ADELSTEIN, A.M. & BULUSU, L. (1984). *Immigrant Mortality in England and Wales, 1970–1978*. OPCS studies on medical and population subjects no 47. HMSO: London.

MCKAY, F.W., HANSON, M.R. & MILLER, R.W. (1982). *Cancer Mortality in the US: 1950–1977*. National Cancer Institute monograph no. 59: Bethesda.

NATIONAL CANCER INSTITUTE (1986). *Cancer among Blacks and Other Minorities: Statistical Profiles*. National Institutes of Health publication number 86-2785: Bethesda.

O.P.C.S. (1981). *International Migration 1980*. Series MN no. 7. HMSO: London.

O.P.C.S. (1983). *Labour Force Survey Monitor*, 22 February. OPCS: London.

PARKIN, D.M. (1986). (ed.). *Cancer Occurrence in Developing Countries*. IARC scientific publication no. 75. IARC: Lyon.

PARKIN, D.M., LAARA, E. & MUIR, C.S. (1988). Estimates of the worldwide frequency of sixteen major cancers in 1980. *Int. J. Cancer*, **41**, 184–197.

PERSAUD, V. (1976). Cancer incidence in Jamaica: an 18 year analysis (1958–75). *W. Ind. Med. J.*, **25**, 201–215.

PERSAUD, V. (1986). Cancer control in the Commonwealth Caribbean. *W. Ind. Med. J.*, **35**, 145–148.

ROSS, R., BERNSTEIN, L., JUDD, H., HANISCH, R., PIKE, M. & HENDERSON, B. (1986). Serum testosterone levels in healthy young black and white men. *JNCI*, **76**, 45–48.

SCHOENBERG, B. (1982). Nervous system. In *Cancer Epidemiology and Prevention*. Schottenfeld, D. & Fraumeni, J.F. Jr (eds.) pp. 968–983. WB Saunders: Philadelphia.

SCHOTTENFELD, D. & WARSHAUER, M. (1982). Testis. In *Cancer Epidemiology and Prevention*. Schottenfeld, D. & Fraumeni, J.F. Jr. (eds). pp. 947–957. WB Saunders: Philadelphia.

VALL MAYANS, M., HALL, A.J., INSKIP, H.M., CHOTARD, J., LINDSAY, S.W., COROMINA, E., MENDY, M., ALONSO, P.L. & WHITTLE, H. (1990). Risk factors for transmission of Hepatitis B virus to Gambian children. *Lancet*, **336**, 1107–1109.

WATERHOUSE, J., MUIR, C., SHANMUGARATNAM, K. & POWELL, J. (1982). (eds). *Cancer Incidence in Five Continents Volume IV*. IARC scientific publication no. 42. IARC: Lyon.

WATSON, J. (1977). *Between Two Cultures: Migrants and Minorities in Britain*. Blackwell: Oxford.